# Effect of Multi-Antioxidant Supplement on Lipid Profile, Occupational Fatigue, Work Stress, and Hair Cortisol in Administrative Workers with and Without Obesity: A Quasi-Experimental Pilot Study

**DOI:** 10.3390/healthcare14091166

**Published:** 2026-04-27

**Authors:** María del Carmen López-García, Gabriel Lara-Hernández, Hamlet Avilés-Arnaut, Virginia Sánchez-Monroy, Eduardo Nateras-Molina, Ernesto Fragoso-Paniagua, Ericka Flores-Berrios, Elvia Pérez-Soto

**Affiliations:** 1 Laboratorio de Biomedicina y Salud Ocupacional, Escuela Nacional de Medicina y Homeopatía, Instituto Politécnico Nacional, Ciudad de México 07320, Mexico; mlopezg@ipn.mx (M.d.C.L.-G.); efragosop2000@alumno.ipn.mx (E.F.-P.); 2 Investigación y Desarrollo, BioMaussan, Ciudad de México 01376, Mexico; gabobmol@gmail.com; 3 Facultad de Ciencias Biológicas, Instituto de Biotecnología, Universidad Autónoma de Nuevo León, Campus Ciudad Universitaria, Av. Universidad S/N, San Nicolás de los Garza 66455, Mexico; hamlet.avilesarn@uanl.edu.mx; 4 Escuela Superior de Medicina, Instituto Politécnico Nacional, Ciudad de México 11340, Mexico; vsanchezm@ipn.mx; 5 Secretaría Académica, Instituto Politécnico Nacional, Ciudad de México 07738, Mexico; enaterasm@ipn.mx; 6 Investigación y Desarrollo, Biodesarrollos Valmex, Tlajomulco de Zúñiga 45640, Mexico; eflores@biovalmex.com

**Keywords:** apple polyphenols, astaxanthin, fucoxanthin, lipid profile, hair cortisol, work stress, burnout, occupational fatigue

## Abstract

**Highlights:**

**What are the main findings?**

- A multi-antioxidant dietary supplement 2.0 with apple polyphenols, astaxanthin, and fucoxanthin reduced occupational fatigue, Burnout syndrome, and work stress scores after 30 days of supplementation.
- Hair cortisol concentration decreased in administrative workers following supplementation, regardless of obesity status.

**What are the implications of the main findings?**

- The observed effects suggest that antioxidant-based nutritional strategies may support stress-related outcomes in occupational health contexts.
- Given the exploratory of this pilot study and the modest lipid changes with limited clinical relevance, the findings should be regarded as preliminary and require confirmation in larger, well-controlled trials.

**Abstract:**

**Background/Objectives**: Work stress (WS), occupational fatigue (OF), and Burnout syndrome (BS) among administrative workers are associated with negative psychosocial and metabolic effects. Although antioxidant-rich nutritional strategies have been proposed to help manage stress, evidence from real-world occupational settings is still limited. This study evaluated the total antioxidant capacity (TAC) of a multi-antioxidant dietary supplement 2.0 (DS2.0; apple polyphenols, [APP], astaxanthin [AXT], and fucoxanthin [FXT]; 387:12:1 ratio) and explored its association with metabolic parameters, OF, psychosocial outcomes, and hair cortisol concentration (HCC) in administrative workers with and without obesity. **Methods**: A quasi-experimental pilot study was conducted among 22 workers, who received DS2.0 (52.13 mg/day, n = 17) or a placebo (n = 5) for 30 days. TAC was analytically assessed using standardized assays. Metabolic outcomes (lipid profile, fasting plasma glucose), psychosocial variables (SOFI-SM, CESQT/SBI, and IMSS tests), and HCC (competitive immunoassay) were evaluated before and after supplementation. Statistical analyses included within-group pre–post comparisons, independent-sample tests, and effect size estimation. **Results**: DS2.0 demonstrated high TAC. Supplementation was associated with reductions in total lipids, total cholesterol, and non-HDL cholesterol, as well as decreases in OF, BS, and WS scores. HCC decreased in the overall sample (217.19 vs. 31.64 pg/mg; *p* = 0.000) and among workers with obesity (276.80 vs. 34.13 pg/mg; *p* = 0.002). Stress-related symptoms, including sleep deprivation, exhaustion, appetite changes, difficulty waking, and palpitations, also improved (*p* ≤ 0.05). **Conclusions**: An antioxidant-rich DS2.0 supplement may be associated with psychosocial and stress-related biomarkers; however, these exploratory findings require confirmation in larger randomized controlled trials. Trial registration: ISRCTN 12762846.

## 1. Introduction

Obesity is a chronic, multifactorial condition and a major global healthcare challenge with significant economic and occupational implications [1,2,3]. In the United States, economic losses amount to 73.1 billion dollars annually due to health problems among full-time workers. According to the World Health Organization (WHO), obesity is a condition characterized by excessive adipose tissue accumulation and commonly assessed using body mass index (BMI). In Mexico, the prevalence of overweight and obesity among adults aged 20 and older is significant, at 38.3% and 36.9%, respectively, according to the 2022 National Health and Nutrition Survey (ENSANUT) [3,4]. In this context, obesity represents a particularly relevant challenge in occupational health settings.

Office and administrative workers represent a particularly vulnerable occupational group, as they are exposed to prolonged sedentary behaviors, limited physical activity, and sustained psychosocial stress [5,6]. These conditions contribute to metabolic dysregulation, altered eating behaviors, fatigue, and chronic activation of stress-response pathways, especially among individuals with obesity [5,6,7]. Evidence suggests a bidirectional relationship between obesity, work stress (WS), and burnout syndrome (BS); however, obesity appears more frequently to precede and exacerbate psychological distress rather than result from it [1,2,3]. Fatigue emerges as a central overlapping symptom linking obesity and psychosocial stress, highlighting the need for integrated preventive approaches.

Occupational fatigue (OF), WS, and BS are considered psychosocial risks that significantly affects workers’ physical and mental health, well-being, and job performance [8,9,10,11]. WS arises when job demands exceed workers’ adaptive capacity and is widely prevalent, with symptoms including fatigue, irritability, sleep disturbances, somatic discomfort, and weight fluctuations [8,11,12,13]. Approximately 70% of individuals respond to stress by overeating or gaining weight, facilitating the development of obesity and related metabolic disorders, and reinforcing the clinical and public health relevance of work-related stress [14].

From a healthcare perspective, challenging work conditions are increasingly linked to heightened oxidative stress (OS), characterized by excessive reactive oxygen species (ROS) and impaired antioxidant defenses. This imbalance leads to cellular damage, energy depletion, reduced ATP synthesis, and persistent fatigue, while promoting metabolic alterations and chronic disease risk [15]. Prolonged exposure to occupational stressors accelerates oxidative damage and cellular aging and has been associated with exhaustion, cognitive decline, and metabolic disorders [15]. Despite these findings, the combined impact of sedentary work, chronic psychosocial stress, OS, and obesity remains insufficiently explored in administrative workers.

WS and BS are also associated with dysregulation of the hypothalamic–pituitary–adrenal axis (HPA), as evidenced by increased hair cortisol concentration (HCC), a validated biomarker of long-term systemic cortisol exposure over weeks to months [11,16]. Elevated HCC has been linked to the pathogenesis of several stress-related conditions, including BS [8,11], anxiety, depression [16], cardiovascular disease, metabolic syndrome, and obesity [2,8].

In this context, recent research highlights the potential role of dietary supplements (DS) with antioxidant and anti-inflammatory properties as supportive strategies to mitigate stress-related metabolic and physiological disturbances. Compounds such as coenzyme Q10, omega-3 fatty acids, vitamin E, carotenoids, including astaxanthin (AXT) and fucoxanthin (FXT), and polyphenols (e.g., tea green and black, red wine, dark chocolate, apples) have demonstrated potential to reduce OS, improve mitochondrial function, and support energy metabolism under high psychosocial stress conditions [15,17,18,19,20,21].

Apple polyphenols (APPs), derived from Malus pumila Mill., are rich in flavonoids, catechins, procyanidins, and phenolic acids, and show strong antioxidant, anti-inflammatory, and hypolipidemic effects [18,19,21,22]. Emerging evidence suggests that APPs may also influence stress-related pathways, including modulation of HPA axis activity, while improving fatigue and performance-related outcomes [21,23]. However, interventional studies evaluating multi-antioxidant formulations targeting WS, metabolic alterations, and HCC remain limited, especially among sedentary administrative workers.

In this work, the DS was formulated with a focus on hydrophilic antioxidants, particularly APPs, which are noted for their superior antioxidant capacity and beneficial effects on OS, lipid metabolism, favorable bioavailability profile, and fatigue-related outcomes [18,19,21,22]. Complementary lower doses of lipophilic carotenoids (AXT and FXT) were included to enhance antioxidant and anti-inflammatory actions, as well as potential modulation of stress-related pathways, while minimizing excessive carotenoid exposure. Accordingly, the 387:12:1 ratio was selected to reflect this hydrophilic–lipophilic balance rather than to represent a pharmacological dose–response optimization, as in some reports [23,24,25].

Given evidence that multi-antioxidant supplementation may provide greater biological benefits than single-compound interventions [24], integrative DS approaches warrant further investigation for their potential to improve well-being in employees exposed to high psychosocial stress and obesogenic environments. Accordingly, this study hypothesized that supplementation with an antioxidant-rich formulation containing APPs, AXT, and FXT (DS2.0) would improve lipid profile parameters, reduce OF, WS and BS, and modulate cortisol levels.

Accordingly, the objective of this quasi-experimental pilot study was to assess the total antioxidant capacity of DS2.0 and to evaluate its anti-lipidemic, anti-fatigue, and anti-stress properties in administrative workers with and without obesity, including an exploratory change in HCC, a biomarker of chronic work-related stress and obesity.

## 2. Materials and Methods

### 2.1. Determination of Total Antioxidant Capacity

#### 2.1.1. Chemicals and Standards

The chemicals and analytical standards listed below were used to determine the total antioxidant capacity (TAC) of the multi-antioxidant dietary supplement 2.0 (DS2.0) and its constituent compounds in vitro. Specifically, these reagents were used in the ferric-reducing antioxidant power (FRAP) assay, the 2,2′-azino-bis (3-ethylbenzothiazoline-6-sulfonic acid) radical cation (ABTS) assay, and the 2,2-diphenyl-1-picryl-hydrazyl (DPPH) free radical scavenging assay. These reagents were employed to generate calibration curves, prepare working solutions, and ensure standardized quantification of TAC. All chemicals were of analytical grade and obtained from certified suppliers.

The chemicals used included 2,4,6-Tri(2-pyridyl)-s-triazine, TPTZ (Cas-No. 3682-35-7) for the FRAP assay; ABTS diammonium salt (Cas-No. 0931-67-0) for the ABTS radical cation assay; and DPPH radical (CAS No. 1898-66-4) for the DPPH free radical scavenging assay. (±)-6-Hydroxy-2,5,7,8-tetramethylchromane-2-carboxylic acid, Trolox (Cas-No. 53188-07-1), was used as the reference antioxidant standard for calibration, while D-α-tocopherol succinate (Cas-No. 4345-03-3) served as a positive control. All chemicals were of analytical grade and were obtained from Sigma-Aldrich (St. Louis, MO, USA). Solutions were freshly prepared according to standardized protocols to ensure analytical reliability and reproducibility.

#### 2.1.2. Multi-Antioxidant DS2.0 and Formulation Components

The evaluated natural compounds (NCs) correspond to APPs extracted from unripe apples (*Malus pumila* Mill.; ApplePhenon^®^), AXT derived from *Haematococcus pluvialis* (AstaZine^®^)*,* and finally FXT extracted from *Laminaria japonica* (ThinOgen^®^). All compounds were supplied by BGG World (Beijing, China, https://bggworld.com/; accessed 5 February 2025). The final DS2.0 formulation, containing APPs, AXT, and FXT in a 387:12:1 ratio, was prepared as a suspension by BioMaussan (Mexico City, Mexico).

The DS2.0 supplement was approved by the Federal Commission for the Protection against Sanitary Risks (COFEPRIS), the Mexican regulatory agency responsible for health product safety.

#### 2.1.3. Determination of TAC by FRAP, ABTS and DPPH Assays

For TAC analysis, the individual NCs were diluted in deionized water to concentrations equivalent to those present in DS2.0 supplement (APPs, 5.03 mg/mL; AXT, 0.17 mg/mL; and FXT, 0.014 mg/mL). The DS2.0 was diluted 1:100 in deionized water to obtain a working concentration of 5.21 mg/mL, preserving the 387:12:1 formulation ratio. D-α-tocopherol succinate (5 mg/mL) and cyclodextrins (5 mg/mL) were used as positive and negative controls, respectively. All measurements were performed in triplicate using a BioTek Epoch spectrophotometer (BioTek Instruments, Inc., Winooski, VT, USA).

TAC values were calculated using a Trolox calibration curve (15.62–1000 μM) and expressed as millimoles of Trolox equivalent antioxidant capacity per milliliter (mM TEAC/mL). The FRAP assay was performed according to a modified Benzie and Devaki method, with absorbance measured at 593 nm [26]. ABTS radical scavenging activity was assessed following the method of Arnao et al., with absorbance read at 754 nm after a 7-min incubation at room temperature [23]. The DPPH assay was conducted by monitoring the decrease in absorbance at 517 nm, reflecting free radical scavenging activity upon interaction with antioxidant compounds [27].

### 2.2. Ethical Statement

This study was conducted in accordance with the Declaration of Helsinki and complied with national regulations for research involving human participants. The protocol was reviewed and approved by the Ethics Committee of the National School of Medicine and Homeopathy, Instituto Politécnico Nacional (IPN) (approval code: CBE/002/2025; approval date: 6 August 2025). The study was registered under ISRCTN 12762846. The registration was performed retrospectively, after patient enrollment had begun, due to administrative delays. All study procedures and outcomes were defined a priori, and the delayed registration does not affect the study’s viability. All participants provided written informed consent before participation.

#### 2.2.1. Study Design

This study was designed as a pilot, exploratory, quasi-experimental intervention to assess the feasibility and short-term effects of a DS2.0 in an occupational setting. The intervention was conducted over 30 days (between June and July 2025) at a private company in Mexico City, Mexico.

Given the pilot nature of the study, the primary objectives were to explore short-term associations between supplementation and selected biochemical, psychosocial, and stress-related parameters, rather than to test confirmatory hypotheses. Although participant allocation to the intervention and placebo groups was initially planned, randomization was compromised due to pragmatic recruitment constraints and participant attrition, resulting in unequal group sizes. Accordingly, the study should be considered a feasibility-oriented pilot investigation rather than a fully randomized controlled trial.

#### 2.2.2. Study Population, Recruitment, and Participants

Participant recruitment was carried out using non-probability convenience sampling. Full-time administrative workers from various departments of a private company in Mexico City were invited to participate through workplace visits conducted by the company physician. All participants had been engaged in in-person administrative work for at least one year before study enrollment.

The inclusion criteria were employment in a fixed work shift, a minimum of one year of work experience, mental health defined as the absence of a self-reported psychiatric or neurological diagnosis and no current use of psychotropic medication, and a BMI ≥ 20 kg/m^2^ according to WHO criteria. Exclusion criteria included the presence of neurodegenerative conditions, pregnancy or lactation, hypothyroidism, diagnosed psychiatric disorders, hair dye use (which may interfere with HCC analysis), and the use of alternative DS.

Initially, 30 administrative workers were assessed for eligibility. Five individuals were excluded for not meeting the inclusion criteria or for declining participation. Consequently, 25 participants were enrolled, with 18 assigned to the DS2.0 intervention group and 7 to the placebo group.

**Figure 1:**
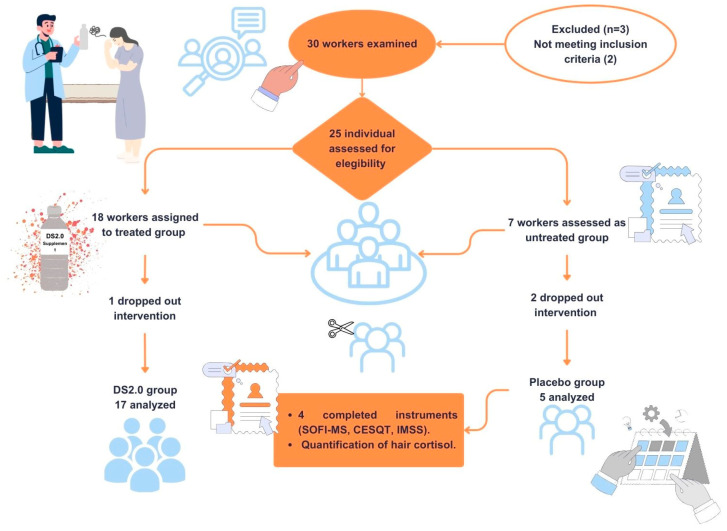
Flowchart of the study population.

During the follow-up period, three participants withdrew from the study, resulting in a final analytical sample of 22 participants. Randomization was compromised due to participant attrition and the limited size of the placebo group. A flow diagram illustrating participant recruitment, allocation, follow-up, and analysis is presented in Figure 1.

#### 2.2.3. Assessment of Baseline Lifestyle Characteristics

Baseline characteristics were evaluated pre-intervention. Data gathered included physical activity, dietary habits, smoking status, alcohol consumption, and history of allergic conditions. Lifestyle variables were documented solely for descriptive analysis and were not modified. Participants were instructed to maintain their usual dietary patterns, physical activity levels, and lifestyle habits throughout the 30-day supplementation. Given the exploratory nature of the study, longitudinal assessments or statistical adjustments for lifestyle variables were not performed; these variables were used to delineate the population and to ascertain confounding factors.

### 2.3. Multi-Antioxidant DS2.0 and Intervention

In the present study, both the DS2.0 supplement and the placebo were administered as suspensions in bottles. The researcher, the medical professional, and all study participants were blinded to the specific composition of both the DS2.0 and the placebo.

Each participant assigned to the intervention group received a dose of 52.13 mg of DS2.0, administered in 10 mL of suspension (composed of 50.4 mg of APPs, 1.6 mg of AXT, and 0.13 mg of FXT, adhering to a 387:12:1 ratio). The daily dose was divided into two administrations, one in the morning and one in the afternoon, over 30 days.

The participants in the placebo group received a suspension containing purified water, citric acid, sorbic acid, allulose, and flavor (Biodesarrollos Valmex, S. A. de C. V., Tlajoculco de Zúñiga, Mexico), and followed the same administration schedule and duration as the DS2.0-supplemented group.

### 2.4. Biochemical Assessments

Blood samples were collected at set time periods on days 1 and 30 of the study. Venous blood was obtained from the brachial vein in the morning after an overnight fast of at least 8–12 h to minimize variability in fasting plasma glucose and lipid measurements. All participants were instructed to refrain from food and caloric beverages during the fasting period before blood collection. Samples were collected using Vacutainer^®^ tubes with red caps (BD, Franklin Lakes, NJ, USA) and centrifuged at 3000 rpm for 10 min at 4 °C. Serum was used to assess FPG, fasting plasma glucose, total lipids (TLs), total cholesterol (TC), triglycerides (TGs), HDL-Cholesterol (HDL), non HDL (non-HDL), LDL-Cholesterol (LDL), VLDL-Cholesterol (VLDL), Atherogenic Index (AI), Urea (U), Blood Urea Nitrogen (BUN), Creatinine (Cr), and Uric Acid (UA). All biochemical analyses were performed by an accredited medical diagnostic laboratory.

### 2.5. Psychosocial Instruments

OF, BS and WS were evaluated pre- and post-intervention via validated questionnaires. OF was measured using the Modified Spanish version of the Swedish Occupational Fatigue Inventory (SOFI-SM) [28], BS with the Spanish Burnout Inventory (CESQT/SBI) [29], and WS through the stress assessment test by the Instituto Mexicano del Seguro Social (IMSS) [30].

The SOFI-SM assesses perceived OF related to physical, mental, and psychological fatigue and is a reliable scale with a Cronbach’s alpha of 0.94 [28], comprising 18 symptoms, grouped into six dimensions: lack of energy, physical tiredness, physical discomfort, drowsiness, lack of motivation, and irritability. The scores obtained help to identify fatigue levels and areas requiring intervention, i.e., acceptable risk level requiring no action (score within 0–25 points), inadequate level requiring advised action (26–50 points), inadequate level requiring priority action (51–75 points) and unacceptable level requiring immediate action (76–100 points) [28].

A validated instrument, CESQT/SBI, was used [29]. This questionnaire comprises 20 items in 4 subscales (illusion for work, psychic exhaustion, indolence, and guilt). The first three subscales allow us to generate an overall burnout score.

Finally, the IMSS test identifies WS and related symptoms, demonstrates reliability (Cronbach’s alpha = 0.9), and evaluates stress-related symptoms using a traffic light scoring system [30]. Symptom intensity is measured on a Likert scale, consisting of 12 items rated on a likert scale. Total scores were classified as follows: no stress (12), no stress but in alarm phase (24), mild stress (36), medium stress (48), high stress (60), and severe stress (72) [30].

### 2.6. Hair Cortisol Concentration Analysis

HCC servs as a biomarker for CS [16]. A 3-millimeter segment of hair was excised from the scalp and preserved at room temperature for analysis. The first centimeter from the hair lock, indicative of stress exposure over the past month, was cut. Subsequently, 10 milligrams of hair were minced, washed with methanol and isopropanol to extract cortisol. The samples were incubated for 16 h at 52 °C, then dried and dissolved in PBS pH 8.0. HCC quantification was performed using a competitive solid-phase enzyme-linked immunosorbent assay (ELISA, ALPCO Diagnostics, Salem, NH, USA, catalogue no. 11-CORHU-E01). We adhere to the manufacturer’s protocol. According to the manufacturer’s technical specifications, the assay has an intra-assay coefficient of variation (CV) ≤8% and an inter-assay CV ≤10%, indicating acceptable analytical precision and reproducibility for research applications. All samples were analyzed in duplicate to further ensure analytical reliability.

HCC in the worker samples (both pre- and post-supplementation, in duplicate) was measured using a microplate reader (Epoch™, BioTek Instruments, Winooski, VT, USA) at 450 nm. HCC was determined from a standard calibration curve.

### 2.7. Adherence to Supplementation

Adherence to the supplementation protocol was encouraged through regular follow-up and supervision by the study physician. Participants received the DS2.0 or placebo in identical containers and were instructed to consume the assigned multi-antioxidant formulation daily according to the prescribed dosing schedule throughout the intervention period. Participants were also advised to maintain their usual dietary habits, physical activity levels, and work routines during the study.

Adherence was evaluated via self-report during follow-up visits; nonetheless, the lack of objective compliance metrics constrained accurate measurement and is recognized as a limitation of this pilot study.

### 2.8. Statistical Analysis

TAC outcomes, including FRAP, ABTS, and DPPH, were analyzed using a one-way ANOVA. When overall group differences were observed, Tukey’s post hoc test was used when appropriate.

Categorical variables were summarized as frequencies and percentages. Quantitative variables, including psychosocial instrument scores, HCC, lipid parameters, and metabolic biomarkers, were reported as mean ± standard deviation or median (range), depending on normality.

Within-group changes from pre- to post-supplementation were assessed using the paired *t*-test or the Wilcoxon signed-rank test, as appropriate, in the DS2.0 group (n = 17) and the placebo group (n = 5). Between-group comparisons were conducted using independent-samples *t*-tests, with Levene’s test applied to assess homogeneity of variances. When distributional assumptions were violated, non-parametric Mann–Whitney U tests were used as confirmatory analyses.

The treated group was additionally stratified by obesity status according to BMI, defining a first subgroup as workers without obesity (BMI ≤ 24.9; including normal weight; n = 4) and a second subgroup as workers with obesity (BMI > 25; including overweight or pre-obesity; n = 13), to explore differential responses to DS2.0 supplementation.

To complement hypothesis testing and aid clinical interpretation, effect sizes were calculated using Cohen’s d. Effect sizes across the analyzed outcomes ranged from trivial to extremely large (approximately −0.04 to 2.7), with the largest and most consistent effects observed for post-intervention HCC. Hedges’ correction and Glass’s delta were examined when appropriate to account for unequal variances or sample sizes. The limited sample size reflects the exploratory nature of the study, which was designed to generate preliminary evidence.

Correlation analyses were conducted for exploratory purposes to describe internal associations within the supplemented group and were not intended to infer intervention effects.

A significance threshold of *p*-value ≤ 0.05 was delineated. Data analysis was performed using the Statistical Package for the Social Sciences (SPSS, Version 27; IBM Corp., Armonk, NY, USA) and GraphPad Prism software (version 8.0.1; GraphPad Software, San Diego, CA, USA).

## 3. Results

### 3.1. Total Antioxidant Capacity in DS2.0 and Constituents

The FRAP, ABTS, and DPPH assays were used to assess the TAC of the NCs and the DS2.0 supplement. Table 1 indicates significant TAC variability among NCs (APPs, AXT and FXT). The DS2.0, composed of APPs, FXT, and AXT in a 387:12:1 ratio, exhibits a notable TAC, correlated with APP’s TAC, yet shows higher TAC by the FRAP assay (*p* ≤ 0.05). The TAC of DS2.0 supplement appears to be largely driven by its APP content, which represents the predominant component of the formulation.

AXT and FXT constituents exhibited lower antioxidant activity than multi-antioxidant DS2.0 in FRAP and DPPH assays.

Our results attribute this to low concentrations of NCs in the supplement. Although APPs show significant TAC, incorporating natural hydrophilic (APP) and lipophilic (AXT and FXT) compounds in varying ratios enhances antioxidant capacity, thereby affecting biological effects.

### 3.2. Baseline Lifestyle and Descriptive Characteristics of the Administrative Workers

Baseline lifestyle characteristics are detailed in Table 2. The study population was predominantly sedentary lifestyle, with participants largely lacking regular physical activity and maintaining consistent dietary habits. The prevalence of alcohol consumption and allergic conditions was low and evenly distributed across groups. Notably, smoking rates were significantly elevated in the placebo group, constituting the principal baseline imbalance identified.

This study involved 22 individuals, with the majority aged 46.45 ± 12 years. Of these, 77.27% (17/22) were female, and 22.72% (5/22) were male.

Furthermore, 27.27% were of normal weight or workers without obesity (6/22), while 22.72% (5/22) were categorized as overweight and 50% as obese, as shown in Table 3.

### 3.3. Short-Term Variations in Lipid Profile Parameters, OF, BS, and WS in Administrative Workers

Evaluation of metabolic health among administrative workers is essential for determining the DS2.0 supplement’s exploratory change on the lipid profile, as well as on OF, BS, and WS scores. Across the study period, most biochemical parameters remained within established reference ranges. In the DS2.0 group, slight improvements were observed in TLs and TC while non-HDL cholesterol levels remained at borderline values, as shown in Table 4. No statistically significant differences were observed between the DS2.0 and placebo groups for lipid or metabolic parameters (*p* > 0.05).

Furthermore, the findings demonstrate a notable difference in initial and final OF, BS and WS scores for the treated group, with *p*-values ≤ 0.05 (Table 4), suggesting a significant enhancement in occupational well-being.

Despite, the DS2.0 group showed significant improvement in occupational well-being, reflected by reductions in OF, BS and WS scores from baseline to post-intervention (*p* ≤ 0.05; Table 4), no significant between-group differences were observed for OF, BS, and WS scores at baseline or post-treatment (all *p* ≥ 0.05); however, no significant between-group differences were observed at baseline or post-intervention. Effect sizes were small and not clinically meaningful, indicating no differential effect versus placebo.

### 3.4. The DS2.0 Supplement Decreased Levels of OF, BS, WS and Stress-Related Symptoms in Administrative Workers

**Figure 2:**
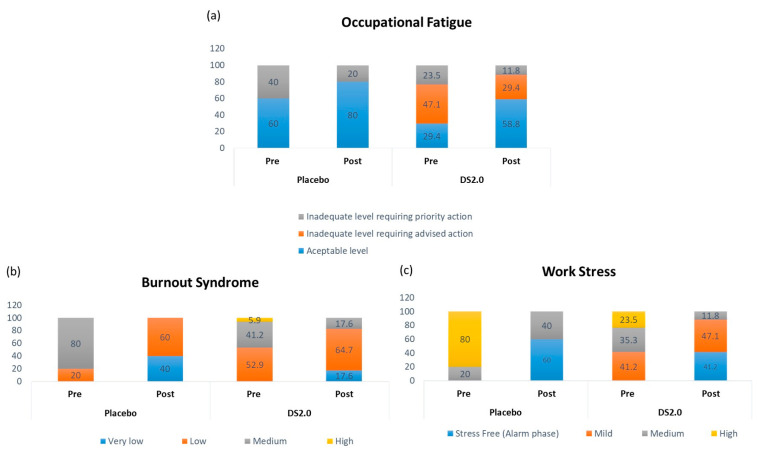
Influence of the DS2.0 supplement on occupational fatigue (OF), burnout syndrome (BS) and work stress (WS) in administrative workers (%). (**a**) Occupational fatigue (OF); (**b**) burnout syndrome (BS). (**c**) work stress (WS). Pre- and post-supplementation percentages are shown for the placebo group (n = 5) and the DS2.0 intervention group (n = 17).

Supplementation with DS2.0 shows a significant change in OF levels. Before supplementation, OF levels were considered inadequate and required advice in 47.1% (8/17), priority action in 23.5% (4/17). Post-supplementation, OF levels notably decreased, indicating stabilization toward more manageable levels, 58.8% of workers reached an acceptable level, as shown in Figure 2a.

In addition, in the DS2.0-supplemented group, the medium level of SB is 41.2% (7/17) and the high level is 5.9% (1/17). Post-supplementation, DS2.0 reached a low BS of 64.7% (11/17). Finally, the high level of SB disappears completely after supplementation, indicating a reduction in severe cases (Figure 2b).

An instrument assessed WS at the symptom level. At the beginning, 23.5% (4/17) of workers had high WS (4/17), followed by 35.3% (6/17) with medium WS and 41.2% (7/17) with mild WS. After supplementation, mild WS was most common among workers (47.2%, 8/17), followed by stress-free (41.2%, 7/17). Finally, the high-stress level decreased to 11.8% (2/17) (Figure 2c).

The reductions in OF, BS, and WS reflect a significant improvement in occupational well-being, suggesting a beneficial exploratory change in the intervention.

Consequently, the exploratory change in multi-antioxidant DS2.0 formulation on physical, cognitive, and behavioral symptoms was evaluated using the IMSS test, which revealed statistically significant reductions in various symptoms in the DS2.0 group (*p*-values ≤ 0.05; Table 5).

### 3.5. Correlation Between OF, WS, and Key Metabolic Indicators in Administrative Workers Supplemented with DS2.0

Exploratory correlation analyses conducted among supplemented administrative workers revealed a moderate positive association between OF and WS, indicating that higher fatigue levels were associated with increased WS. In addition, TG levels were inversely correlated with HDL cholesterol, and HDL cholesterol was negatively correlated with AI, consistent with established cardiometabolic risk patterns (see Table 6).

These correlations are presented descriptively and should be interpreted as exploratory, given the pilot design and limited sample size.

### 3.6. Safety and Tolerability

Safety was assessed via clinical follow-up and structured inquiries about adverse effects. No significant adverse events or interruptions in the study were noted. In the DS2.0-supplemented group, one participant experienced mild gastrointestinal discomfort (5.8%, 1/17) that resolved on its own and required no medical assistance. The placebo group reported no adverse events.

Renal function parameters were consistent before and after supplementation in each group. Safety monitoring lacked a standardized adverse-event grading system and formal statistical analysis, reflecting the exploratory design and limited sample size. Consequently, while the results indicate short-term tolerability of the multi-antioxidant DS2.0 formulation, the potential for rare, subtle, or delayed adverse effects cannot be excluded.

## 4. Discussion

Research indicates that OF, BS, and WS are increasingly common among employees [10,11]. These conditions can adversely affect health, leading to metabolic problems such as obesity [2], whose prevalence continues to rise [2,4].

Consequently, a comprehensive, multidisciplinary approach is essential for managing metabolic disorders associated with WS to enhance employee well-being [2,3]. Such approaches may include nutritional strategies aimed at supporting metabolic health and psychosocial outcomes; however, their effectiveness and underlying mechanisms require further investigation [2,3]. In this context, the present pilot study aims to assess the feasibility of a multi-antioxidant DS2.0 formulation for biochemical parameters, OF, BS, and WS among administrative workers, and to conduct an exploratory assessment of HCC.

As an initial step, the TAC of the DS2.0 formulation was assessed. DS2.0 consists of APPs from unripe apples, AXT, and FXT in a 387:12:1 ratio, resulting in a formulation predominantly composed of hydrophilic compounds and with low concentrations of lipophilic carotenoids. DS2.0 exhibited a higher TAC than α-tocopherol and the individual natural compounds (AXT, FXT, and apple polyphenols), underscoring the antioxidant potential of the formulation. These findings are consistent with previous studies reporting antioxidant activity for AXT [15], FXT [15], polyphenols, in apple varieties [31], vegetable juices [29], and supplements containing combinations of hydrophilic and lipophilic compounds [24].

Although the elevated TAC suggests a strong antioxidant profile, the present study was not designed to determine whether the observed effects reflect specific synergistic interactions among components or are primarily driven by APPs, which constitutes the major fraction of the formulation. Therefore, the combined formulation showed high TAC across assays; however, interaction effects among components were not specifically evaluated. The inclusion of cyclodextrins enhances compound stability and solubility [32], supporting formulation consistency and analytical reliability.

Baseline descriptive analyses indicated a mean BMI of 30.12 ± 7.25, with a high prevalence of excess body weight among administrative workers. Overweight and obesity were present in 72.7% of participants, driven mainly by obesity (50%), a pattern consistent with national ENSANUT 2022 data reporting an 84.6% prevalence of overweight/obesity among Mexican adults aged 40–59 years [4]. This background highlights the coexistence of metabolic vulnerability and WS in this population.

**Figure 3:**
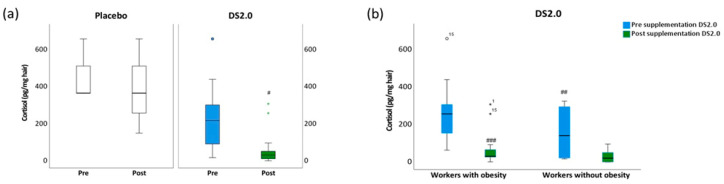
Influence of the DS2.0 supplementation on hair cortisol concentration (HCC) in administrative personnel. (**a**) HCC in study groups, placebo (n = 5), and DS2.0 supplementation (n = 17). (**b**) HCC in the DS2.0 group stratified by obesity status: workers with obesity (n = 12) and workers without obesity (n = 5). Nonparametric tests were applied for variables with non-normal distributions, with statistical significance set at *p* ≤ 0.05. (#) Pre- vs. post-supplementation comparisons were performed using the Wilcoxon signed-rank test; (##) Comparison between workers with and without obesity was conducted using the Mann–Whitney U test. (###) Pre- vs. post-supplementation comparisons within obesity subgroups were performed using the Wilcoxon signed-rank test. Data are presented as box plots showing the median, interquartile range, and potential outliers. Asterisks (*), circles, and numbers denote outlier values identified in the analysis.

Before supplementation, OF was reported by 76.5% (12/17) of participants and was predominantly associated with physical and mental fatigue, a prevalence comparable to that documented in professional environments involving computer-mediated and repetitive tasks [9]. All participants demonstrated WS and BS, signifying compromised occupational well-being at baseline. Elevated HCC were observed (Figure 3a), particularly among workers with obesity (257.70 pg/mg; 70.58%), consistent with prior evidence that correlates CS contexts with populations exhibiting suboptimal nutritional status [33]. These findings underscore the importance of cortisol as a marker of CS exposure in this population, although causal relationships cannot be inferred within the current methodological framework, particularly given the potential influence of unmeasured biological, occupational, and lifestyle-related factors.

Following 30 days of supplementation, significant within-group reductions were observed in OF, WS, and BS scores, accompanied by improvements in stress-related manifestations, including insomnia, fatigue, altered appetite, morning lethargy, and palpitations. These psychosocial enhancements coincided with a pronounced reduction in HCC, particularly among workers with obesity, suggesting attenuation of hypothalamic–pituitary–adrenal (HPA) axis activity. Given the cumulative nature of HCC as a marker of long-term stress exposure, the magnitude of the observed decline should be interpreted cautiously; however, the consistency of this finding across analyses supports its potential relevance [11,33,34]. At the same time, interindividual variability in cortisol regulation, obesity-related metabolic alterations, occupational characteristics (e.g., cognitive workload, work-shift, sedentary behavior, work organization), psychosocial stressors, and lifestyle habits such as sleep quality and dietary patterns are likely to contribute to the observed heterogeneity in responses [1], and should be considered potential confounders.

The interpretation of HCC findings must also consider the regulatory framework of NOM-035-STPS-2018 [35], which utilizes organizational and perceptual methods for risk assessment, excluding biomarkers. Consequently, HCC is considered here solely as a complementary research indicator rather than a diagnostic or regulatory tool. While regulatory application is beyond the scope of this study, the cortisol-related findings suggest that biomarkers such as HCC could potentially complement psychosocial evaluations in more comprehensive occupational health assessments, pending validation in larger studies.

Previous studies have reported associations between dietary antioxidants and stress-related and metabolic outcomes. Polyphenols have demonstrated anti-stress effects and reductions in cortisol levels, alongside improvements in OS pathways [36,37,38]. Likewise, carotenoids such as lutein, zeaxanthin, and astaxanthin have been linked to improvements in psychological stress and cortisol-related measures in selected models and interventions [39,40,41]. Collectively, these findings suggest that antioxidant-rich nutritional strategies may influence stress regulation; however, extrapolation to the present results remains speculative, particularly given uncontrolled occupational and lifestyle-related confounders.

Consistent with the pilot nature of the study, between-group analyses revealed no statistically significant differences between the placebo and DS2.0 groups for lipid parameters or psychosocial outcomes. Observed lipid changes were modest, imprecise, and of limited clinical relevance, with small-to-moderate effect sizes and wide confidence intervals. This pattern likely reflects limited statistical power, unequal group allocation, and residual confounding, as commonly reported in exploratory nutritional intervention studies [21,42,43].

In contrast, HCC exhibited more consistent within-group reductions and larger effect sizes, suggesting that stress-related biological systems may respond more rapidly to short-term nutritional modulation than traditional metabolic markers. Chronic cortisol exposure has been shown to influence sleep quality, perceived fatigue, emotional depletion, and metabolic dysregulation, providing a plausible biological substrate for the psychological improvements [11,13,34]. Nonetheless, the absence of direct OS or enzymatic measurements precludes attribution to specific antioxidant-mediated or endocrine mechanisms.

From a translational perspective, future analysis should explore integrated strategies targeting CS, obesity, and metabolic vulnerabilities, while accounting for changes in HCC and lipid metabolism. Pharmacological approaches focused on the 11β-hydroxysteroid dehydrogenase type 1 (11β-HSD1) pathway have emerged as a promising strategy for obesity management [13], with evidence that resveratrol, a polyphenolic antioxidant, inhibits 11β-HSD1 activity in adipose tissue and liver [44]. However, in the absence of direct OS and enzymatic measurements, the present findings cannot be attributed to specific mechanisms, such as modulation of cortisol metabolism or 11β-HSD1 activity.

Psychosocial outcomes were assessed via self-reported instruments, which are inherently susceptible to expectancy and placebo effects, especially in pilot intervention studies lacking full blinding. Meta-analytic evidence suggests self-reported outcomes are more influenced by expectation than objective physiological measures, necessitating cautious interpretation of subjective improvements [45]. Furthermore, significant placebo responses have been frequently documented in psychological and stress-related conditions [46], underscoring the need to consider expectancy effects when interpreting changes in perceived OF, WS, and BS.

Nevertheless, concordance between reductions in perceived stress and modulation of hair cortisol concentration strengthens internal coherence. Overall, these results are hypothesis-generating and support the feasibility of integrating biochemical, psychosocial, and neuroendocrine measures in workplace research. From an occupational health perspective, nutritional strategies warrant further evaluation within integrated stress and fatigue management programs [1,5,34]. Larger randomized trials with biomarkers of OS, cortisol metabolism assessment, and longitudinal follow-up are required to confirm mechanisms and sustainability effects.

## 5. Strengths and Limitations of the Study

This pilot study has several limitations that should be considered when interpreting the findings. First, the use of non-probability convenience sampling may have introduced selection bias and limited representativeness and generalizability. Although this approach is common in exploratory research and offers valuable preliminary insights, it inevitably reduces validity.

In Table 1, baseline lifestyle attributes were comparable between groups; however, a significant imbalance in smoking status emerged, potentially creating residual confounding. Furthermore, lifestyle variables were evaluated by self-report at baseline only and were not included as covariates in statistical analyses due to the limited sample size and pilot design. Differences in working conditions between groups, including shift work, overtime, and additional employment, may have further contributed to unmeasured confounding, particularly in stress-related outcomes.

Statistical analyses relied primarily on within-group comparisons, as the study was not powered for time × group interaction models and group sizes were unequal, which explains the absence of significant between-group effects.

In contrast, HCC demonstrated more pronounced and consistent changes, with marked within-group reductions and large effect sizes, despite the absence of formal between-group interaction testing. While this pattern suggests a potentially meaningful effect on stress-related biological pathways, it must be interpreted with caution, given the pilot design and limited sample size.

Exploratory BMI-based subgroup analyses and HCC outliers further reflect biological variability rather than intervention effects.

Although adherence and adverse effects were monitored through clinical follow-up and physician-led interviews, the absence of objective adherence measures and a standardized adverse event grading system limits the precision of compliance and safety estimates. Importantly, no clinically relevant alterations were observed in the assessed biochemical parameters, suggesting that the intervention was well tolerated from a metabolic and biochemical safety perspective. Collectively, these considerations support the interpretation of findings as hypothesis-generating within an exploratory feasibility study.

## 6. Conclusions

DS2.0, an apple polyphenol-rich antioxidant supplement, was evaluated in this quasi-experimental pilot study, demonstrating the feasibility of integrating biochemical, psychological, and HCC assessments in administrative workers. Although no significant between-group differences were observed and lipid-related changes were of limited clinical relevance, consistent within-group reductions in HCC and psychosocial stress indicators suggest a stress-modulating effect. Larger, adequately powered trials are required to confirm clinical relevance and underlying mechanisms.

## 7. Impact

Despite the absence of significant placebo-controlled effects, this pilot study highlights the feasibility of integrative stress assessment in the workplace and suggests that DS2.0 may exert a potential stress-modulating effect, warranting confirmation in larger, adequately powered trials.

## Figures and Tables

**Table 1:** Total antioxidant capacity (TAC) of the Multi-Antioxidant Dietary Supplement (DS2.0) and Natural Compounds (NCs) (Mean ± SD, n = 3).

DS, NCs/ Dilution	FRAP (mM TE/mL)	ABTS^•+^ (mM TE/mL)	DPPH^•^ (mM TE/mL)
DE (1:0)	0.0000 ^e^	0.0000	0.0000
AT (1:2)	0.8211 ± 0.0655 ^e^	0.3135 ± 0.0446 ^a,c,d^	1.6750 ± 0.0110
AXT (1:100)	0.6783 ± 0.0123 ^e^	0.0035 ± 0.0036 ^b^	0.0075 ± 0.0037
FXT (1:100)	0.2506 ± 0.0072 ^e^	0.0021 ± 0.0042 ^b^	0.0050 ± 0.0036
APP (1:100)	78.6100 ± 0.6905 ^a,b,c,d^	39.7600 ± 0.1100 ^a,b,c,d^	31.0700 ± 0.2068 ^a,b,c,d^
DS2.0 (1:100)	81.3500 ± 2.5450 ^a,b,c,d,e^	39.7600 ± 0.0492 ^a,b,c,d^	33.1700 ± 2.1010 ^a,b,c,d^

Results are expressed as the mean ± SEM from triplicate assays. The final values were expressed as mean millimoles of Trolox equivalents (TE) per milliliter (mM TE/mL). All natural compounds (NCs) and DS2.0 were diluted at 1:100 unless otherwise indicated. Superscripts letters indicate statistically significant differences as follows: (a) Different from cyclodextrin (DE); (b) Different from α-tocopherol (AT); (c) different from astaxanthin (AXT); (d) different from Fucoxanthin (FXT); (e) different from Apple polyphenols (APPs). Statistical analysis was performed using one-way ANOVA followed by appropriate post hoc testing (Graph Pad Prism). Differences were considered significant at *p* ≤ 0.05. Abbreviations: DE, cyclodextrin; AT, α-tocopherol; AXT, astaxanthin; FXT, fucoxanthin (FXT); APPs, apple polyphenols; DS2.0, multi-antioxidant dietary supplement 2.0 (marine algae-derived formulation). ABTS^•+^ refers to the radical cation of 2,2′-azino-bis(3-ethylbenzothiazoline-6-sulfonic acid) used in the antioxidant capacity assay. DPPH^•^ refers to the stable free radical 2,2-diphenyl-1-picrylhydrazyl used in the antioxidant activity assay.

**Table 2:** Baseline Lifestyle and Health-Related Characteristics of the Study Population.

Variable	Total (n = 22)	Placebo (n = 5)	Intervention (n = 17)	*p*-Value †
**Physical activity, n (%)**				
Performs physical activity	6 (27.3)	1 (20.0)	5 (29.4)	1.000
Does not perform physical activity	16 (72.7)	4 (80.0)	12 (70.6)	
**Weekly exercise frequency (days/week), n (%)**				
0 days	16 (72.7)	4 (80.0)	12 (70.6)	0.885 ‡
≥1 day	6 (27.3)	1 (20.0)	5 (29.4)	
**Daily meal frequency, n (%)**				
2 meals/day	7 (31.8)	1 (20.0)	6 (35.3)	0.774
3 meals/day	12 (54.5)	3 (60.0)	9 (52.9)	
>3 meals/day	3 (13.6)	1 (20.0)	2 (11.8)	
**Smoking status, n (%)**				
Smoker	7 (31.8)	4 (80.0)	3 (17.6)	0.021
Nonsmoker	15 (68.2)	1 (20.0)	14 (82.4)	
**Weekly cigarette consumption, n (%)**				
Does not smoke	16 (72.7)	2 (40.0)	14 (82.4)	0.145
≥1 cigarette/week	6 (27.3)	3 (60.0)	3 (17.6)	
**Alcohol consumption, n (%)**				
Yes	3 (13.6)	0 (0.0)	3 (17.6)	1.000
No	19 (86.4)	5 (100.0)	14 (82.4)	
**Frequency of alcohol consumption, n (%)**				
No consumption	19 (86.4)	5 (100.0)	14 (82.4)	1.000
2–3 times/week	3 (13.6)	0 (0.0)	3 (17.6)	
**Allergies, n (%)**				
Present	7 (31.8)	2 (40.0)	5 (29.4)	1.000
Absent	15 (68.2)	3 (60.0)	12 (70.6)	

Data are presented as the number of participants (percentage). All variables correspond to baseline assessments and were not interventional outcomes. † *p*-values were calculated using Fisher’s exact test or chi-square test, as appropriate. Due to the small sample size, Fisher’s exact test was preferred when expected cell counts were <5. ‡ Spearman correlation was used for ordinal variables (weekly exercise frequency).

**Table 3:** Descriptive Characteristics of the Administrative Workers.

Variable	Total (n = 22)	Placebo (n = 5)	DS2.0 (n = 17)
Sex, n (%)			
Female	7 (77.27)	5 (100)	12 (70.60)
Male	5 (22.73)	-	5 (29.40)
Age (years) ^1^	46.45 ± 12.64	47.40 ± 14.58	46.18 ± 11.95
Weight (kg) ^1^	79.90 ± 17.00	63.56 ± 12.86	82.02 ± 17.68
Height (m) ^1^	1.60 ± 0.08 ^1^	1.56 ± 0.08	1.61 ± 0.08
BMI Categories, n (%)			
Normal weight	6 (27.30)	2 (40)	4 (23.52)
Overweight	5 (22.70)	2 (40)	3 (17.64)
Obesity class I	4 (18.18)	-	4 (23.52)
Obesity class II	5 (22.72)	1 (20)	4 (23.52)
Obesity class III	2 (9.09)	-	2 (11.76)
BMI (kg/m^2^)	30.12 ± 7.25	5 (26.07)	17 (31.31)
Years of service (years)	17.7 ± 8		
Shift, n (%)			
Morning	19 (86.36)	5 (100)	14 (82.40)
Mixed	3 (13.64)	-	3 (17.60)
Overtime, n (%)			
Yes	8 (36.36)	-	8 (47.10)
No	14 (63.63)	5 (100)	9 (52.90)
Additional work, n (%)			
Yes	3 (17.60)	-	3 (17.60)
No	19 (82.40)	5 (100)	14 (82.40)

Data are presented as the number of participants (percentage), unless otherwise indicated. ^1^ For continuous variables, values are expressed as mean ± standard deviation. BMI, Body mass index.

**Table 4:** Effect of DS2.0 on Biochemical Parameters and Psychosocial Scales in Administrative Workers.

Variable		Placebo n = 5	*p* Value	DS2.0 n = 17	*p* Value	Reference Range
FPG	Pre Post	82 (78–83) 91 (83–94)	0.0421	84 (65–254) 95 (84–161)	0.0531	70–99, normal
TLs	Pre Post	526.00 ± 97.92 501.80 ± 117.49	0.1252	602.93 ± 76.18 569.60 ± 89.54	0.0500	400–800, normal
TC	Pre Post	167 (162–288) 170 (150–284)	0.2251	221.33 ± 31.89 208.60 ± 40.17	0.0182	<200, normal 200–239, borderline high
TGs	Pre Post	130.40 ± 40.90 115.80 ± 33.41	0.2332	160.26 ± 77.02 152.40 ± 63.94	0.5482	<150, normal 150–199, borderline high
HDL	Pre Post	38.2 ± 13.33 36.80 ± 45.0	0.4542	37.93 ± 8.63 36.20 ± 7.61	0.2652	>40 (men), >50 (women), protective
Non-HDL	Pre Post	159.60 ± 45.00 156.20 ± 44.66	0.3682	183.40 ± 28.89 172.40 ± 37.77	0.0342	130–159, moderate risk 160–189, elevated risk, often requires medical intervention
LDL	Pre Post	133.52 ± 48.53 133.04 ± 44.99	0.9112	151.34 ± 36.22 141.92 ± 41.46	0.0982	130–159, moderate risk
VLDL	Pre Post	26.08 ± 8.18 23.16 ± 6.68	0.2332	32.05 ± 15.40 30.48 ± 12.78	0.5482	5–40, normal
AI	Pre Post	5.25 ± 0.88 5.35 ± 0.76	0.6892	6.02 ± 1.25 5.93 ± 1.46	0.7332	>4.5, elevated cardiovascular risk
Urea	Pre Post	37.82 ± 5.74 33.62 ± 6.48	0.2312	37.09 ± 8.94 35.40 ± 6.98	0.2122	15–40 mg/dL, normal
BUN	Pre Post	17.67 ± 2.68 15.71 ± 3.02	0.2312	17.33 ± 4.17 16.54 ± 3.26	0.2122	7–20 mg/dL, normal
Cr	Pre Post	0.93 ± 0.09 0.91 ± 0.13	0.5262	0.93 ± 0.18 0.92 ± 0.19	0.4242	~0.6–1.3, normal
UA	Pre Post	5.0 ± 0.79 4.94 ± 1.09	0.7992	5.89 ± 1.38 5.94 ± 1.32	0.7302	~3.5–7.0, normal
OF	Pre Post	43 (34–96) 35 (19–62)	0.2251	41.7 ± 17.3 26.2 ± 15.9	0.0052	26–50, inadequate level requiring advised action, 51–75, inadequate level requiring priority action
BS	Pre Post	36.0 (36–40) 29.0 (2–32)	0.1091	27.0 (16–58) 20.0 (11–48)	0.0011	11–33, low BS 34–66, medium BS
WS	Pre Post	34.20 ± 13.59 25.60 ± 10.26	0.3422	37.5 ± 11.1 27.5 ± 8.70	0.0012	24–35, no stress but in alarm phase; 36–47, mild stress; 48–59, medium stress; 61–72, severe

Statistical significance was defined as *p* ≤ 0.05. Biochemical variables are reported in mg/dL. Psychological variables are reported as scale scores (points) using the corresponding validated instruments. Reference ranges are based on ADA and WHO guidelines. Abbreviations: FPG, Fasting plasma glucose; TLs, total lipids; TC, total cholesterol; TGs, triglycerides; HDL, high-density lipoprotein; non-HDL, non-high-density lipoprotein; LDL, low-density lipoprotein; VLDL, very-low-density lipoprotein; AI, atherogenic index; BUN, blood urea nitrogen; Cr, creatinine; UA, uric acid; OF, occupational fatigue; BS, burnout syndrome; WS, work stress.

**Table 5:** Effect of DS2.0 Supplement on Stress-Related Symptoms (WS) in the Administrative Workers.

Variable		Placebo (n = 5)	*p* Value	DS2.0 (n = 17)	*p* Value
Inability to sleep	Pre	3 (2–3)	0.564	4 (1–6)	0.005
	Post	2 (1–4)		3 (1–5)	
Headaches and migraines	Pre	3 (2–3)	0.083	3 (1–5)	0.249
	Post	4 (3–4)		2 (1–4)	
Indigestion or gastrointestinal discomfort	Pre	3.0 ± 1.7	0.667	3.35 ± 1.5	0.681
	Post	3.3 ± 1.1		3.18 ± 1.5	
Feeling of extreme tiredness or exhaustion	Pre	4.0 ± 2.0	0.426	3.88 ± 1.4	0.007
	Post	3.7 ± 2.5		2.76 ± 1.2	
Tendency to eat, drink, or smoke more than usual	Pre Post	4 (2–6) 2 (2–6)	0.317	3 (1–6) 2 (1–6)	0.150
Decreased sexual interest	Pre Post	2 (1–2) 1 (1–2)	0.184	3 (1–6) 2 (1–5)	0.204
Shortness of breath or choking sensation	Pre Post	1 (1–2) 1 (1–3)	0.655	2 (1–6) 1 (1–6)	0.072
Appetite	Pre Post	2 (1–2) 1 (1–3)	0.317	3 (1–4) 1 (1–4)	0.031
Muscle tremors	Pre Post	1 (1–5) 1 (1–5)	0.317	2 (1–5) 2 (1–5)	0.158
Prickling or painful sensations	Pre Post	3 (1–6) 2 (1–4)	0.317	3 (1–6) 2 (1–4)	0.420
Strong temptation to feel tired in the morning	Pre Post	4 (1–6) 2 (1–6)	0.414	4 (1–6) 2 (1–6)	0.015
Sweating or palpitations	Pre Post	2 (1–6) 1 (1–6)	0.317	2 (1–6) 1 (1–6)	0.017

Nonparametric data are presented as median (minimum and maximum) and were analyzed using the Wilcoxon signed-rank test. Parametric data are presented as mean ± standard deviation and were analyzed using the paired *t*-test. Statistical significance was defined as *p* ≤ 0.05.

**Table 6:** Exploratory Correlations Between Psychological Outcomes and Key Metabolic Indicators among Supplemented Administrative Personnel (n = 17/22).

Variables	Correlation	Correlation Coefficient, *p*-Value
↓ Occupational Fatigue	↓ Work Stress	0.617 *, 0.014
↓ Triglycerides	↑ HDL cholesterol	−0.637 *, 0.011
↑ HDL cholesterol	↓ Atherogenic index	−0.590 *, 0.021

Data correspond to exploratory correlation analyses among participants receiving DS2.0 (n = 17). Correlation coefficients were calculated using Spearman’s rank correlation test. * Correlation is significant at the 0.05 level (two-tailed). Arrows indicate the direction of change: ↑ denotes an increase and ↓ denotes a decrease in the corresponding variable.

## Data Availability

The datasets generated can be obtained upon request by sending an email to elvperezs@ipn.mx.

## Abbreviations

The following abbreviations are used in this manuscript:

DS2.0	Dietary supplement 2.0
OF	Occupational fatigue
BS	Burnout syndrome
WS	Work Stress
APPs	Apple polyphenols
AXT	Astaxanthin
FXT	Fucoxanthin
TAC	Total antioxidant capacity
HCC	hair cortisol concentration
NCs	natural compounds
CS	Chronic stress
FRAP	Ferric-Reducing Antioxidant Power
ABTS	2,2′-Azino-bis (3-ethylbenzothiazoline-6-sulfonic acid) diammonium salt
DPPH•	(±)-6-Hydroxy-2,5,7,8-tetramethylchromane-2-carboxylic acid
COFEPRIS	Federal Commission for the Protection against Sanitary Risks
AT	D-α-tocopherol succinate
DE	Cyclodextrin
SOFI-SM	Modified Spanish version of the Swedish Occupational Fatigue Inventory
CESQT/SBI	Questionnaire for the Evaluation of the Work Burnout Syndrome/Spanish Burnout Inventory
IMSS test	Work stress test measured by the Instituto Mexicano del Seguro Social
